# Immunogenicity and Serological Cross-Reactivity of Saliva Proteins among Different Tsetse Species

**DOI:** 10.1371/journal.pntd.0004038

**Published:** 2015-08-27

**Authors:** Xin Zhao, Thiago Luiz Alves e Silva, Laura Cronin, Amy F. Savage, Michelle O’Neill, Barbara Nerima, Loyce M. Okedi, Serap Aksoy

**Affiliations:** 1 Yale School of Public Health, Department of Epidemiology of Microbial Diseases, New Haven, Connecticut, United States of America; 2 National Livestock Resources Institute, Tororo, Uganda; IRD/CIRDES, BURKINA FASO

## Abstract

Tsetse are vectors of pathogenic trypanosomes, agents of human and animal trypanosomiasis in Africa. Components of tsetse saliva (sialome) are introduced into the mammalian host bite site during the blood feeding process and are important for tsetse’s ability to feed efficiently, but can also influence disease transmission and serve as biomarkers for host exposure. We compared the sialome components from four tsetse species in two subgenera: subgenus *Morsitans*: *Glossina morsitans morsitans* (*Gmm*) and *Glossina pallidipes* (*Gpd*), and subgenus *Palpalis*: *Glossina palpalis gambiensis* (*Gpg*) and *Glossina fuscipes fuscipes* (*Gff*), and evaluated their immunogenicity and serological cross reactivity by an immunoblot approach utilizing antibodies from experimental mice challenged with uninfected flies. The protein and immune profiles of sialome components varied with fly species in the same subgenus displaying greater similarity and cross reactivity. Sera obtained from cattle from disease endemic areas of Africa displayed an immunogenicity profile reflective of tsetse species distribution. We analyzed the sialome fractions of *Gmm* by LC-MS/MS, and identified TAg5, Tsal1/Tsal2, and Sgp3 as major immunogenic proteins, and the 5'-nucleotidase family as well as four members of the Adenosine Deaminase Growth Factor (ADGF) family as the major non-immunogenic proteins. Within the ADGF family, we identified four closely related proteins (TSGF-1, TSGF-2, ADGF-3 and ADGF-4), all of which are expressed in tsetse salivary glands. We describe the tsetse species-specific expression profiles and genomic localization of these proteins. Using a passive-immunity approach, we evaluated the effects of rec-TSGF (TSGF-1 and TSGF-2) polyclonal antibodies on tsetse fitness parameters. Limited exposure of tsetse to mice with circulating anti-TSGF antibodies resulted in a slight detriment to their blood feeding ability as reflected by compromised digestion, lower weight gain and less total lipid reserves although these results were not statistically significant. Long-term exposure studies of tsetse flies to antibodies corresponding to the ADGF family of proteins are warranted to evaluate the role of this conserved family in fly biology.

## Author Summary

Insect saliva contains many proteins that are injected into the mammalian host during the blood feeding process. Saliva proteins enhance the blood feeding ability of insects, but they can also induce mammalian immune responses that inhibit successful feeding, or modulate the bite site to benefit pathogen transmission. Here we studied saliva from four different tsetse species that belong to two distant species groups. We show that the saliva protein profiles of different species groups vary. Experimental mice subjected to fly bites display varying immunological responses against the abundant saliva proteins and the antigenicity of the shared saliva proteins in different tsetse species differs. We show that one member of the ADGF family with adenosine deaminase motifs, TSGF-2, is non-immunogenic in *Glossina morsitans* in mice, while the same protein from *Glossina fuscipes* is highly immunogenic. Such species-specific immune responses could be exploited as biomarkers of host exposures in the field. We also show that short-term exposure of *G*. *morsitans* to mice passively immunized by anti-TSGF antibodies leads to slight but not statistically significant negative fitness effects. Thus, future investigations with non-antigenic saliva proteins are warranted as they can lead to novel mammalian vaccine targets to reduce tsetse populations in the field.

## Introduction

Tsetse flies are vectors of pathogenic trypanosomes, which cause Human African Trypanosomiasis (HAT), also known as Sleeping Sickness. In west and central Africa, the parasite *Trypanosoma brucei gambiense* causes a chronic but nearly always fatal disease, while in east of the Rift valley, *Trypanosoma brucei rhodesiense* causes an acute disease that is rapidly fatal if untreated [1]. Devastating epidemics in the 20^th^ century resulted in tens of thousands of deaths in sub-Saharan Africa [2]. WHO has recently reported that epidemics that devastated Africa since 1980s have come under control, with case numbers declining below 10,000 for the first time in 2009 [3]. Many HAT endemic countries, including Central African Republic, Chad, Congo, Côte d’Ivoire, Uganda and Sudan, with disease occurring in remote areas, have limited access to surveillance, treatment and control measures [4]. In countries such as Guinea, the first country affected by HAT epidemics in West Africa, surveillance activities were eliminated especially in the context of the EBOLA epidemic. In addition to HAT, nagana or Animal African Trypanosomosis (AAT), caused by *Trypanosoma brucei brucei* and the related parasites, *Trypanosoma congolense* and *Trypanosoma vivax*, limits effective cattle rearing across ten million square kilometers of Africa [5] and has wide implications for land use, agricultural practices and nutrition [6].

Natural transmission of the parasite to the mammalian host requires the insect tsetse host (genus *Glossina*). Based on geographic distribution, behavioral, molecular and morphological characteristics, the genus *Glossina* is split into three species complexes: subgenera *Fusca*, *Morsitans* and *Palpalis* [7]. The *Palpalis* group consists of the major HAT transmitting species associated with forest galleries and thickets along riverine ecosystems, including *Glossina palpalis palpalis (Gpp)* and *Glossina palpalis gambiensis* (*Gpg*) in west Africa; and *Glossina fuscipes* spp. in Democratic Republic of Congo, northern Angola, southern Congo, western Tanzania and Kenya, Uganda, Rwanda, Burundi, and southern Sudan. The *Morsitans* group consists of vectors of HAT and AAT in east and central Africa, including two closely related species, *Glossina morsitans morsitans (Gmm)* and *Glossina pallidipes (Gpd)*, both associated with savannah type ecosystems [8–10].

The role of saliva has been widely documented for successful blood feeding of insects. Analysis of the secreted salivary gland (SG) proteins present in the saliva (termed sialome) of different blood sucking insects have identified functionally conserved molecules that disarm host hemostasis and inflammatory/immune processes. Based on transcriptomic and proteomic approaches the major tsetse SG proteins are known to include the anticoagulant thrombin inhibitor (tsetse thrombin inhibitor, TTI), two putative adenosine deaminases (tsetse salivary growth factors 1 and 2; TSGF-1 and TSGF-2), salivary apyrase (5’ nucleotidase-related SG protein 3; Sgp3), antigen5-related allergen (tsetse Antigen 5; TAg5) and two putative endonucleases (tsetse SG proteins 1 and 2; Tsal1 and Tsal2) [11–18]. Because the often fast evolving insect saliva proteins can be species specific with unique immunogenic properties, the potential use of saliva antigens as biomarkers for host exposure to different tsetse species has been recently investigated [19–22].

In addition to enabling successful blood feeding, sialome proteins also influence pathogen transmission processes at the bite site. For infections caused by sand fly transmitted *Leishmania* spp., fly saliva has been shown to increase lesion size and parasite burden, and enhance the infection rate [23–26]. In the case of *Rhodnius prolixus*, which transmit *Trypanosoma cruzi*, parasite infection is enhanced by immunosuppressant mechanisms of the reduviid bug saliva [27–29]. Tsetse saliva also facilitates *T*. *brucei* infection in mice, possibly resulting from reduced host inflammatory responses [30]. Given the critical role sialome proteins can play in the infection outcome, vaccinating the mammalian host against saliva proteins has been suggested as a means to reduce pathogen transmission, or host feeding ability [31, 32].

Here we compared the major sialome proteins from four tsetse species that belong to two different subgenera: subgenus *Palpalis* (*Gpg* and *Gff*), and subgenus *Morsitans* (*Gmm* and *Gpd)*. *Gpg* and *Gff* are among the most important human disease transmitting tsetse species, while *Gmm* and *Gpd* prefer non-human hosts. We characterized the immunogenic components of the sialome, and determined the serological cross-reactivity that major saliva proteins exhibit between the different species complexes. We focused on the abundant protein family TSGF and characterized the genomic aspects of this family in different tsetse species. Finally, we evaluated the potential use of TSGF proteins as mammalian vaccine antigens to reduce tsetse fitness through passive-immunity approach in experimental mice.

## Material and Methods

### Ethics statement

Mouse experiments were carried out in strict accordance with the recommendations of the Care and Use of Laboratory Animals of the National Institutes of Health. All of the animals were handled according to Yale University Institutional Animal Care and Use Committee (IACUC) approved Protocols 2011–07266 and 2014–07266 (2011–07266 renewed on June 27, 2014). The cattle sera from Uganda were collected by the National Livestock Resources Institute (NaLIRRI) Veterinary team. Prior to the collections, the protocols were developed by NALIRRI Institutional Animal Care and Use Committee and were submitted to and approved by the Uganda National Council for Science and Technology (UNCST) as specified in Reference Number HS 1061, Dec. 1, 2011. The veterinary team obtained the required permission for obtaining the cattle sera from individual owners.

### Animals

Six week old male C57BL/6 mice were used for all experiments. In each case mice were individually housed. *Gmm* (Westwood) are maintained in the insectary at Yale University. Puparia from *Gff*, *Gpg* and *Gpd* were imported from the Institute of Zoology laboratory at Slovak Academy of Science according to USDA Research Permit 30355 to S.A. All flies were maintained at 25°C with 50–55% relative humidity, and received defibrinated bovine blood every 48 h using an artificial membrane system [33].

### SG and saliva contents

SG dissections and saliva collection were performed as previously described [34] with some modifications. Three days after receiving their last blood meal, flies were immobilized and SGs were microscopically dissected and pooled in ice cold sterile PBS (phosphate-buffered saline, 137 mM NaCl, 2.7 mM KCl, 2.4 mM KH_2_PO_4,_ 10 mM Na_2_HPO_4_, pH 7.4). After 1 hour of incubation on ice, all samples were spun down at 2300 g for 10 min, and the supernatant was collected and identified as sialome.

### Generation of anti-*Gmm*, and anti-*Gff* sialome antibodies

Six week old C57BL/6 mice were sedated and exposed to 7–10 tsetse bites 3 times/week for 3 weeks. Two weeks after the final tsetse exposure, blood was collected via cardiac puncture and allowed to clot at room temperature for 20 min, after which samples were centrifuged for 15 min at 600 g at room temperature. The serum fraction was removed and stored at -20°C for long-term storage or 4°C while in use. The sera from five mice exposed to the same tsetse species were combined for immunoblot analysis.

### Recombinant saliva proteins and anti-recProtein antibodies

The recombinant (rec) TSGF-2 protein expression and anti-sera has been described [13]. To generate additional recProteins, the coding sequences of *tsal1*, and *tsgf1* were amplified without the signal peptide region from *Gmm* SG cDNA. The primers used in the amplification process were for *tsal1* (Forward: 5’- CTATGAGCTCTCGTTAAAAATACCAGAGAG and Reverse: 5’- CTCAGCGGCCGCATTAAATTTTAACAAATTATTA); and for *tsgf1* (Forward: 5’- GTACGGATCCGAAGTGAACAAAGCTTATC and Reverse: 5’- GTACCTCGAGTTTCTCCTTCTTTCAAG). PCR amplification products were cloned into the pET-28a vector (Novagen), and transfected into *Escherichia coli* BL21 strain for expression. recTsal1 (43 kD), and recTSGF-1 (54 kD) proteins were purified using the His bind purification kit (Novagen, Cat # 70239–3). Purified recTsal1 and recTSGF-1 were analyzed by SDS-PAGE and the protein bands were sliced and 500 μg protein was used with adjuvant to generate polyclonal sera in rabbits commercially (Cocalico Biologicals, Inc).

### SDS Polyacrylamide Gel Electrophoresis (PAGE) analysis and immunoblotting

Same amount of total sialome proteins obtained from dissected SG (or extracts from the same number of dissected salivary glands) were analyzed by 12% SDS-PAGE under reducing conditions and either stained by coomasie blue, or transferred to nitrocellulose membranes (BioRad, Cat # 162–0112) according to standard protocols [35]. Protein concentration was detected by Nanodrop 2000 Spectrophotometer (Thermo Scientific, Cat# ND2000PR14). For immunoblot analysis, the concentrations of the primary antibodies used were: 1:200 for anti-*Gmm* and anti-*Gff* saliva, 1:10,000 for anti-recTsal1; 1:20,000 for anti-recTSGF-1 and 1:5,000 for anti-recTSGF-2. The secondary antibody goat anti-mouse IgG (H+L)-HRP conjugate (BioRad, Cat # 170–6516) and goat anti-rabbit IgG (H+L)-HRP conjugate (BioRad, Cat # 170–6515) were diluted 1:20,000 before use and SuperSignal west pico chemiluminescent substrate (Thermo Scientific, Cat# 34080) was added for detection and visualized using Molecular Imager ChemidocTM XRS+ (BioRad, Cat #170–8265) following the manufacturer’s instructions. In addition, saliva collected from different tsetse species were separated on 10% native PAGE analysis at 4°C with Tris buffer (25 mM Tris, 200mM glycine) [17] using molecular weights ranging from 14,000–500,000 for non-denaturing PAGE analysis (Sigma_Aldrich, Cat# MWND500) for size estimation. For immunoblot, the proteins were transferred to nitrocellulose membrane using transfer buffer without SDS and the membranes were blotted as described above.

### Immunoblot analysis with cattle sera

Sera were obtained from cattle maintained in the Kibuku and Manafwa districts in south-eastern Uganda. The location and age of the cattle from which sera were collected are listed in S1 Table. Given that individual animal responses and/or exposure to tsetse bites could vary widely in the natural state, we chose to combine sera from 5–6 cattle in the same age group for immunoblot analysis. Sera obtained from cattle used to maintain the *Gff* colony at The National Livestock Resources Research Institute (NaLIRRI), Tororo were used as positive control for exposure. Negative control serum used was commercially obtained FBS (Sigma, Cat # 12105). For immunoblot analysis primary antibodies were diluted 1:2,000 and the secondary antibody goat anti-bovine IgG (Thermo Scientific, Cat # PA1-28700) was diluted 1:20,000 before use.

### LC-MS/MS protein identification

*Gmm* sialome components were separated on 12% SDS PAGE, stained by commassie blue and the visible abundant protein bands were sliced and separated into two fractions. The first fraction contained the protein bands identified as immunogenic based on Immunoblot analysis with anti-saliva antibodies generated in mice. The second fraction contained protein bands identified as non-immunogenic in the Immunoblot analysis. The protein components of both fractions were subjected to LC-MS/MS analysis at W.M. Keck Facility at Yale University. Briefly, peptides were separated on a Waters nanoACQUITY (75 μm x 250 mm eluted at 300nl/min) with MS analysis on a LTQ Orbitrap mass spectrometer. Mascot distiller and the Mascot search algorithm were used for searching in the NCBI database. Confidence level was set to 95% within the MASCOT search engine for protein hits based on randomness.

### Genome and transcriptome data and bioinformatics analysis

Genome data from *Gmm*, *Gpd*, *Glossina austeni (Gau)*, *Gff* and *Glossina brevipalpis (Gbr)* were obtained from Vectorbase (https://www.vectorbase.org/). For SG transcriptome, we used both EST and Illumina data [36, 37]. Transcriptome denovo assembly and mapping were analyzed using CLC Genomics Workbench (CLC bio, Cambridge, MA). Blast, genome annotation and sequence alignment were performed by CLC Main Workbench (CLC bio, Cambridge, MA). The published *Gmm* TSGF sequences and *Drosophila melanogaster* (*Dm*) sequences were used to identify homologs in other *Glossina* species by Blast. The ADA motif associated with each homolog was verified by BlastP analysis. Signal peptides were predicted by SignalP (http://www.cbs.dtu.dk/services/SignalP/). Phylogenic trees were generated using CLC Main Workbench. Jukes Cantor method was used to measure the protein distance and Neighbor Joining method was used to generate the tree. Bootstrap analysis was performed by 1000 replicates.

### Tsetse feeding on passively immunized mice, engorgement and lipid measurements

Four mice were injected intraperitoneally with a total of 400 μg purified IgG (ImmunoPure (A/G) IgG Purification Kit, Pierce, Cat# 44902), corresponding to 200 μg of anti-*Gmm* recTSGF-1 and recTSGF-2 IgG, respectively. Control mice (n = 4) were similarly injected with 400 μg of purified pre-immune rabbit IgG. Rabbit IgG antibody titers were assessed from each mouse by ELISA 24 h, and 12 days after IgG transfer. Two 96 well plates were coated (18 h, 4°C) with 10 μg/ml of purified recTSGF-1 or TSGF-2 in 50μl of coating buffer (0.05 M Na_2_CO_3_, 0.05 M NaHCO_3_, pH 9.6), respectively. Plates were washed five times with 200 μl washing buffer (PBS, pH 7.4, containing 0.1% (v/v) Tween 20) and were then blocked with blocking buffer (5% milk in PBST) for 2 h at room temperature. Plates were washed again and serum samples from mice were 1:1250 diluted and 50μl were added in duplicate wells for each dilution. Rabbit anti- recTSGF-1 or anti-recTSGF-2 antibodies were included as positive control, and normal mice sera were used as negative control. Samples and controls were incubated for 1.5 h at room temperature. For secondary antibody HRP-conjugated goat anti-rabbit IgG (1:5,000) in blocking buffer was added and plates were incubated for 1 h at room temperature. Following 5 washes, 50 μl chromogenic substrate (TMB) were added to each well, plates were incubated for 8 min, and the reaction was terminated with 50 μl H_2_SO_4_ stop solution and absorbance at 450 nm was measured with reduction at 630 nm using ELISA plate reader.

Newly emerged virgin male and female flies (n = 64 male and 64 female) were separated into 16 individual cages with 8 female or male flies per cage. One cage of male flies and one cage of female flies were each randomly assigned to either a TSGF passively immunized mouse or a control mouse, such that the same 16 flies (8 male and 8 female) were fed on only their assigned mouse for the duration of the experiment. Each cage of flies was weighed 24 h before they received their first blood meal and then again immediately after they fed on mice. Flies were exposed to the same mouse at 2, 5, 8 and 11 days post antibody transfer, and allowed to feed for 15 min following the IACUC approved protocols. On each blood meal, one cage of male and one cage of female flies were allowed to feed on one mouse. For detecting engorgement variation, the combined weight of flies (n = 8) in each cage (n = 16) was measured before and after each blood meal on mice to an accuracy of 0.1 mg. Total weight change was also measured by comparing the weight of flies 24 h before the first blood meal and 72 h after the 4th blood meal. The fly survival data was recorded every three days over the experimental period.

At the end of the experiment, total lipid levels were determined using a vanillin assay as previously described [38]. Briefly, flies were collected 72 h after their 4th blood meal on mice, and allowed to dry at 0% RH (relative humidity) at 60°C. Individual flies were homogenized in 0.5 ml of chloroform:methanol (2:1) and 0.1ml of the supernatant was moved into a 5 ml glass tube and the solvent was fully evaporated at 90°C. 0.4 ml of concentrated sulfuric acid was added into the dried lipid and heated at 90°C for 30 min. 4 ml vanillin reagent was added to the acid/lipid mixture. Samples were measured spectrophotometrically at 525 nm, and total lipid content was calculated against a lipid standard (canola oil) [38, 39]. Statistical analyses were performed using the Mann Whitney test in the GraphPad Prism 6 software package.

## Results

### Sialome protein profiles reflect host species relationships

**Fig 1:**
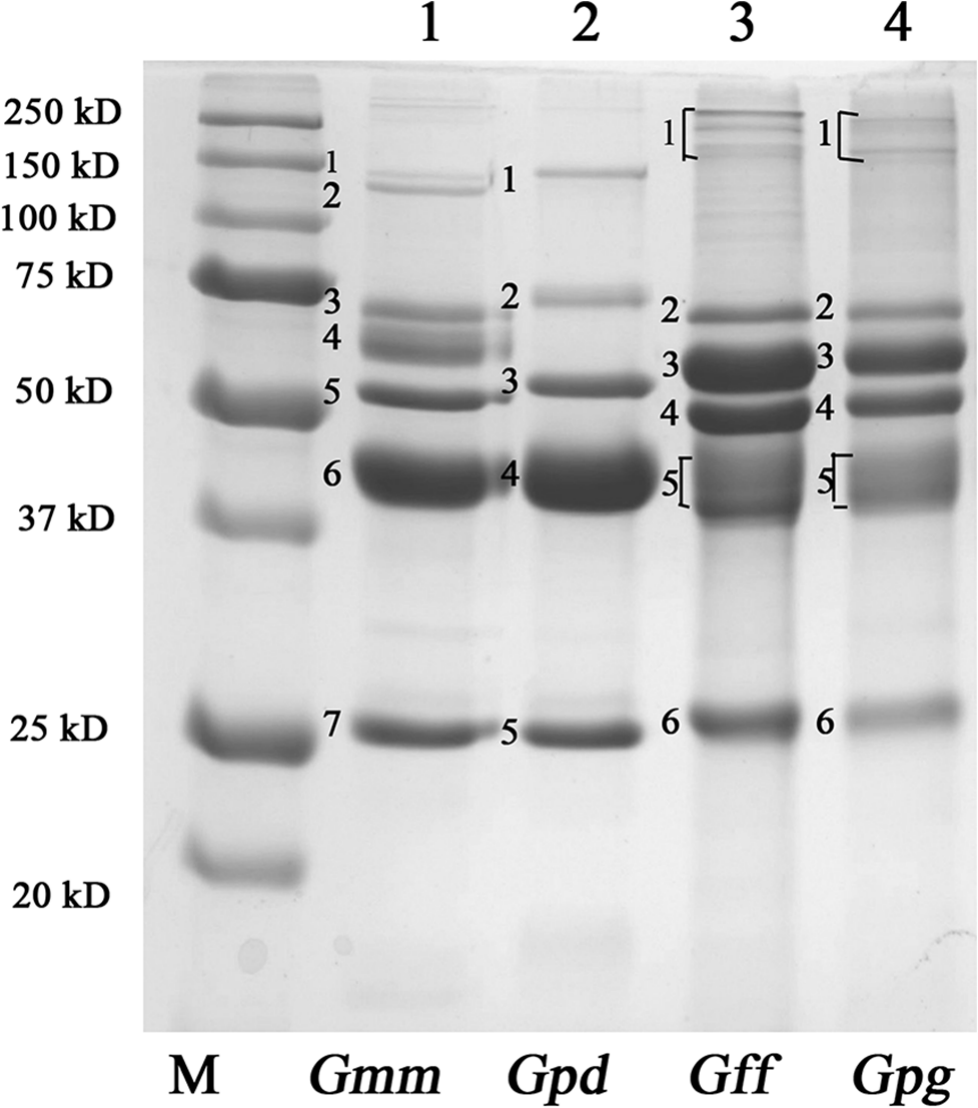
SDS PAGE analysis of saliva from different tsetse species. Lanes 1–4 show protein profiles of *Gmm*, *Gpd*, *Gff* and *Gpg* sialomes analyzed by SDS-PAGE analysis stained by Coomassie Blue. M indicates the Molecular Weight marker. Bands referred to in the sialome of each species are numbered from top to bottom. One representative image for the sialome data is shown. Additional results from SG extracts and replicate sialome samples are shown in S1 Fig.

We analyzed the protein profiles of the sialomes as well as SG extracts from *Gmm*, *Gpd*, *Gff* and *Gpg* by SDS-PAGE analysis (Fig 1 and S1 Fig). Based on staining intensity, sialomes contain several abundant protein fractions, and based on banding profiles, the sialomes of the *Morsitans* group species (*Gmm* and *Gpd)* are more similar to one another than to the species in the *Palpalis* group (*Gpg* and *Gff*). Comparison of the sialome components of the *Morsitans* group species shows three protein fractions of similar sizes (Fig 1, Lane 1 labeled 5–7 in *Gmm*, and Lane 2 labeled 3–5 in *Gpd*, respectively). In addition, two high molecular weight proteins of about 150 kD in size in *Gmm* (Lane 1, bands 1 and 2) can be reproducibly detected, while *Gpd* has a single protein band around this size (Lane 2, band 1) and *Gmm* has a protein (band 3) that runs slightly higher in size than in *Gpd* (band 2). *Gmm* sialome also has an approximately 60 kD protein fraction (labelled 4), which is reproducibly absent from *Gpd*. The overall profiles of the *Gff* and *Gpg* sialome proteins are also similar to one other, but differ from the *Morsitans* group both in size and relative abundance (Lanes 3 and 4). There are several protein bands in *Gff* and *Gpg* sialomes of around 200 kD in size (Lanes 3 and 4, band 1, respectively) in addition to five major highly reproducible protein fractions. The most abundant protein fraction in the *Morsitans* group is about 40 kD in size (Lanes 1 and 2, bands 6 and 4, respectively), while the most abundant sialome protein of *Palpalis* species is about 60 kD in size (band 3 in Lanes 3 and 4). Our results suggest the sialome protein profile is comparable between the species of the two groups, *Morsitans* and *Palpalis*.

### Sialome antibodies from *Morsitans* and *Palpalis* group do not exhibit serological cross-reactivity

**Fig 2:**
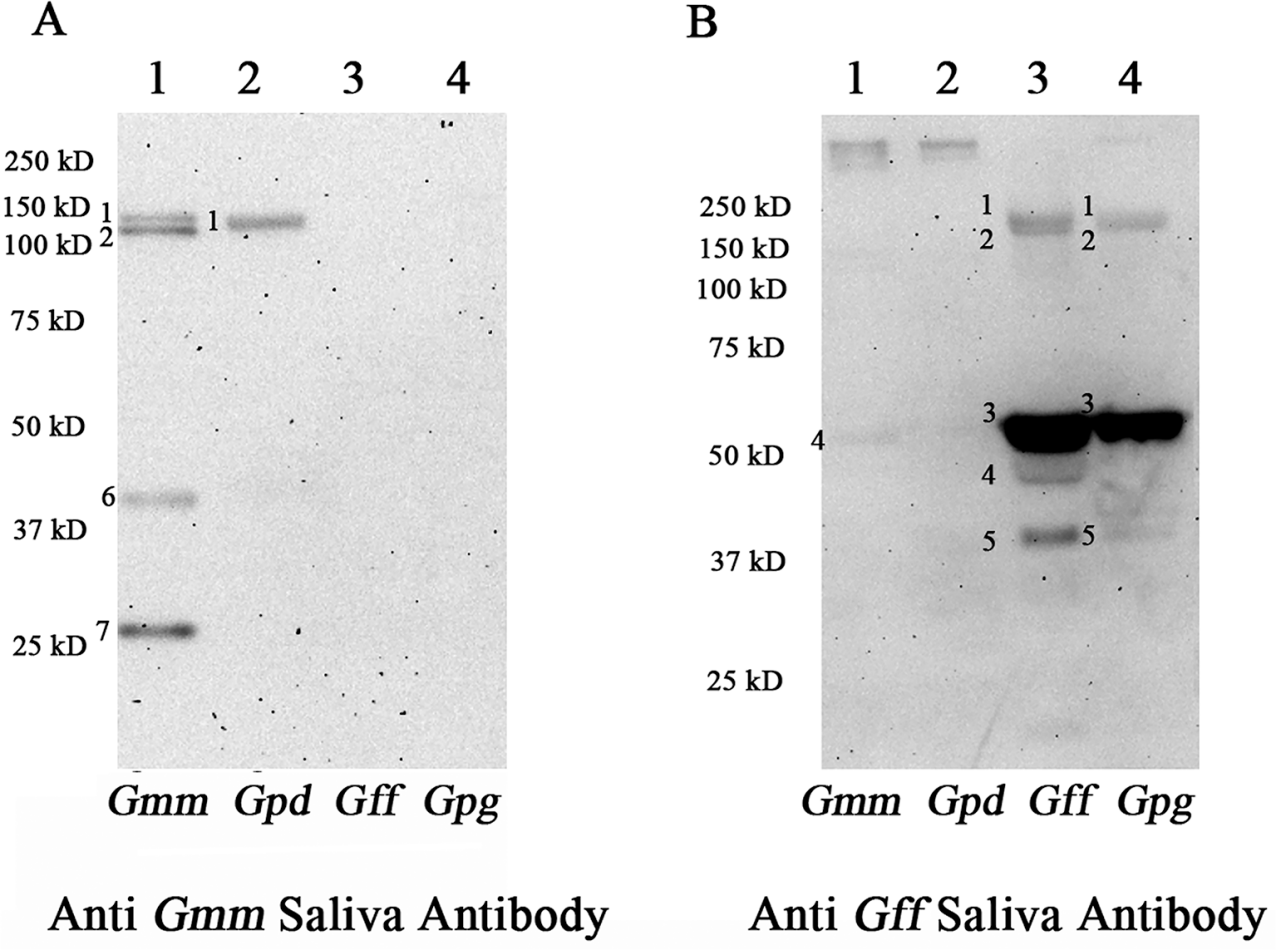
Immunogenic potential of sialome components in different tsetse species. The same amount of sialome proteins from *Gmm*, *Gpd*, *Gff* and *Gpg* were analyzed by immunoblot analysis using sera obtained from mice exposed to (A) *Gmm* and (B) *Gff* bites. Bands detected are numbered corresponding to Fig 1.

To determine the antigenic potential of the sialome proteins, we performed immunoblotting with anti-*Gmm* and anti-*Gff* saliva antibodies generated in mice, respectively (Fig 2). *Gmm* anti-saliva antibodies consistently recognized four major protein fractions in *Gmm* (Fig 2A, Lane 1): two proteins of about 150 kD (bands 1 and 2), 40 kD (band 6), and 25 kD (band 7) in size. In contrast, the same *Gmm* anti-saliva antibodies recognized only one protein of about 150 kD in *Gpd* sialome (Fig 2A, Lane 2, band 1). Anti-*Gmm* saliva antibodies did not show serological cross reactivity with either *Gff* or *Gpg* sialomes (Lanes 3 and 4, respectively). We next used anti-*Gff* saliva antibodies to analyze the *Gmm* and *Gpd* sialome preparations using the same immunoblotting approach. We detected reliably only two large proteins (>250 KB in size) from *Gmm* and *Gpd* sialomes (Fig 2B, Lanes 1 and 2, respectively), although very weak hybridizing bands of around 55 KD and two bands of around 150K were also detected. By contrast, anti-*Gff* saliva antibodies detected multiple proteins from the *Gff* sialome (Fig 2B, Lane 3); two high molecular weight protein fractions (band 1 and 2), and three proteins ranging in size from 40–55 kD (bands 3–5 in Lane 3), and generated a similar profile with *Gpg* sialome, except that band 4 was not detectable and the intensity of band 5 was less pronounced (Lane 4). The immunoblots were repeated using different sialome preparations and with anti-saliva antibodies generated in different mice. These results collectively showed the *Gmm*, *Gpd* and *Gpg* species-specific profiles to be highly reproducible (S2 Fig). Native PAGE analysis under non-denaturing conditions and subsequent immunoblot analysis were also performed (S3 Fig). Similar to the results obtained under denaturing conditions, anti-*Gmm* saliva antibodies detected one major protein band in *Gmm* and *Gpd* sialomes analyzed under non-denaturing conditions, while no cross-reactivity was noted with *Gpg* sialome (S3B Fig). The anti-*Gff* saliva antibodies detected one strong and several less pronounced bands in the *Gpg*, and only a single weak band in the *Gpd* sialome, but no cross-reactivity was noted with *Gmm* sialome (S3C Fig).

### Immunogenicity of proteins in *Gmm* sialome

**Fig 3:**
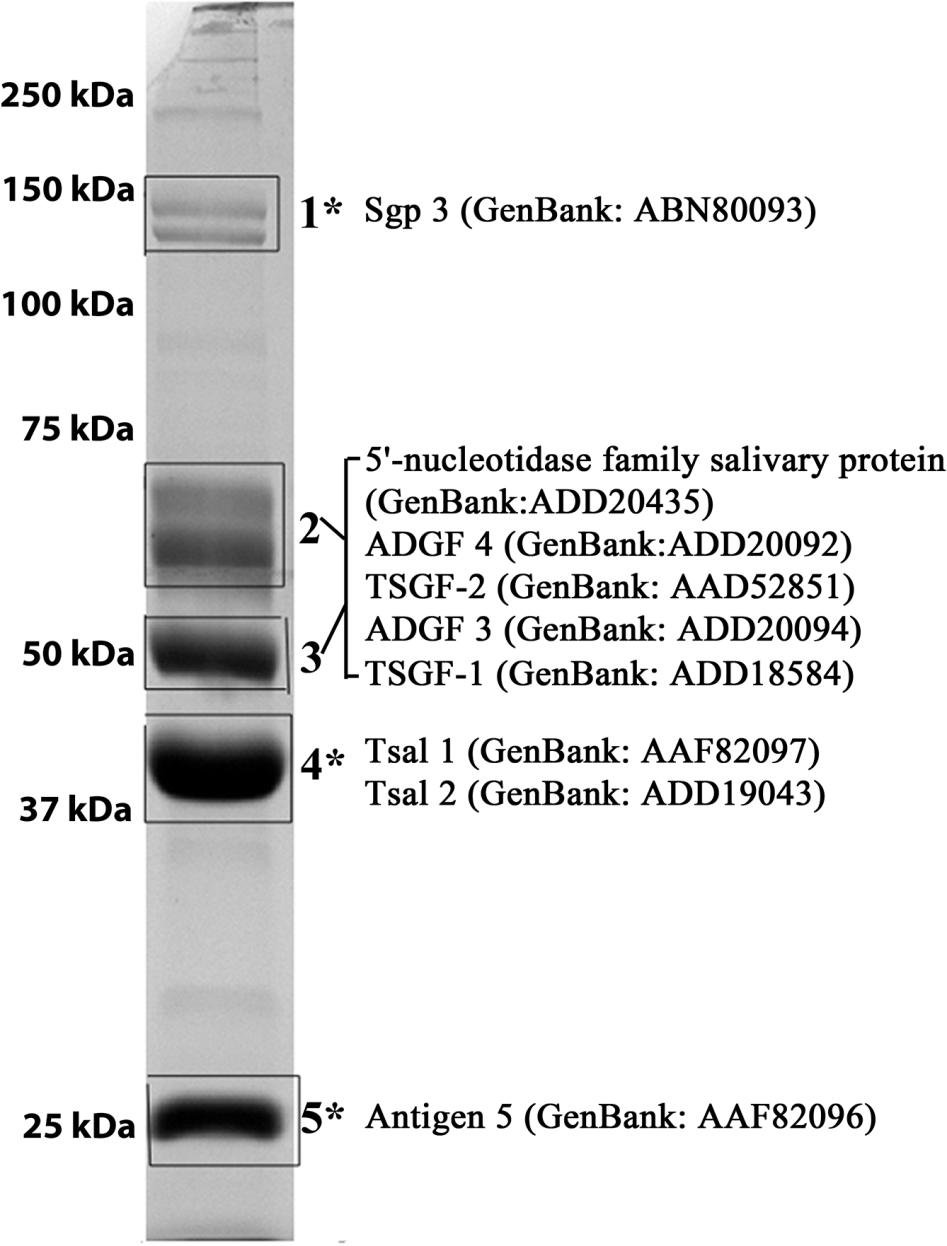
Identification of the major immunogenic and non-immunogenic proteins in *Gmm* sialome. The most abundant protein bands extracted for LC-MS/MS analysis are shown boxed. Fractions 1, 4 and 5, as marked by “*”, correspond to protein bands that were noted as immunogenic in the analysis presented in Fig 2. Protein fractions 2 and 3 were combined representing non-immunogenic proteins.

To identify the immunogenic and non-immunogenic components of the sialome, we combined the *Gmm* sialome proteins into two fractions as “immunogenic” and “non-immunogenic” based on our immunoblot analysis (Fig 3, bands 1, 4 and 5 versus bands 2 and 3, respectively). We subjected the two fractions to LC-MS/MS analysis and used the *Gmm* transcriptome database to predict the putative peptides present in each fraction [36]. This analysis identified the immunogenic proteins as TAg5, Tsal1/Tsal2, and Sgp3, while the non-immunogenic fraction included the 5'-nucleotidase family and four members of the Adenosine Deaminase Growth Factor (ADGF) family (Fig 3, S1 Table). We had previously characterized two members of the ADGF family, TSGF-1 and TSGF-2 [13], as abundant proteins in the salivary glands. The other two members of the ADGF family, Salivary Secreted Adenosine (Genbank Number ADD20094) and Adenosine Deaminase-related-Growth Factor C (Genbank Number ADD20092), were previously annotated in the *Gmm* database as ADGF-3 and ADGF-4, respectively [40].

### Recombinant protein antibodies recognize sialome proteins across species complexes

**Fig 4:**
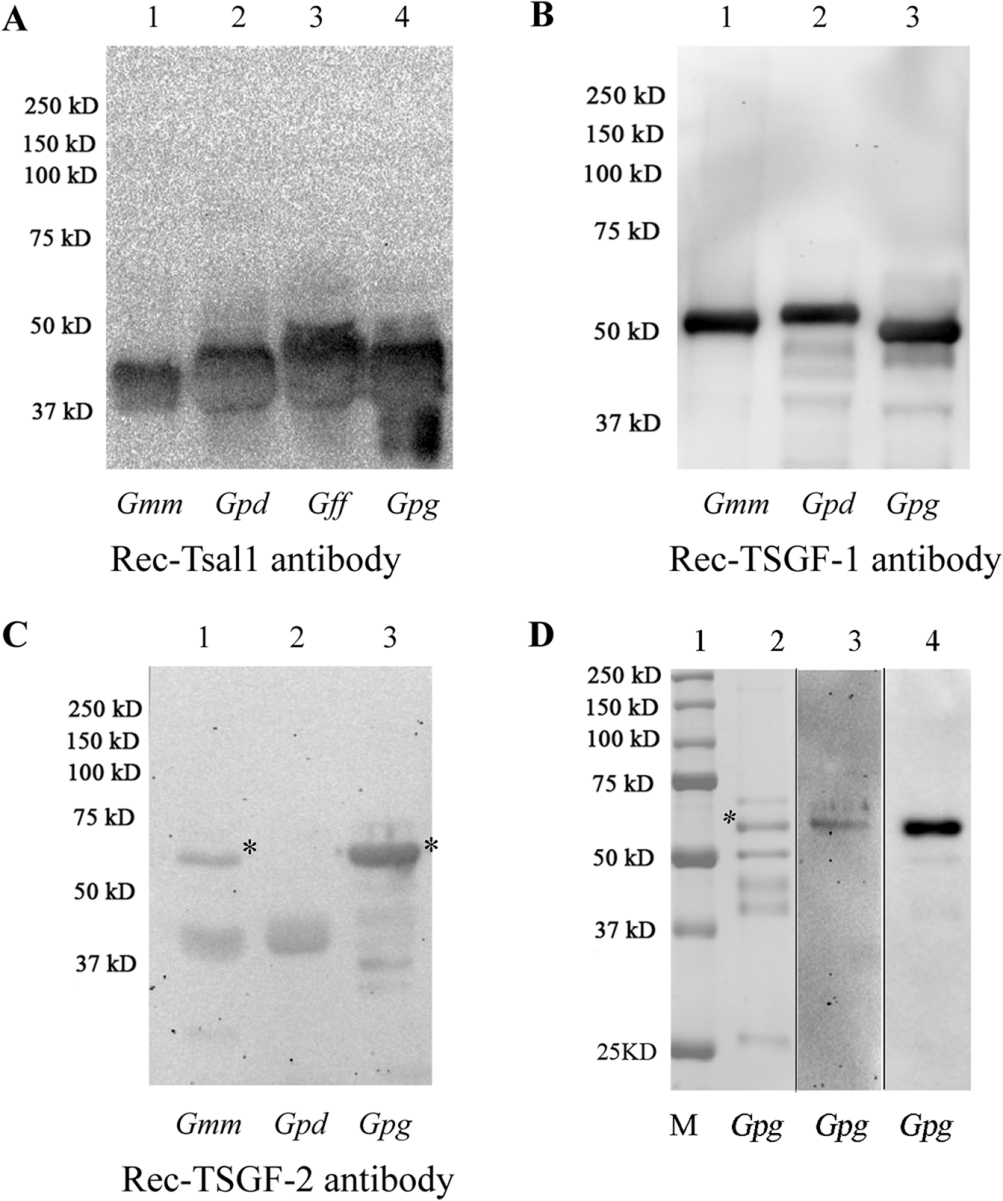
Immunoblot of sialome proteins from *Gmm*, *Gpd*, *Gff* and *Gpg*. Same amount of sialome proteins from *Gmm*, *Gpd*, *Gff* and *Gpg* were probed with (A) rec-*Gmm* Tsal1, (B) rec-*Gmm* TSGF-1 and (C) rec-*Gmm* TSGF-2 antibodies, respectively. (D) *Gpg* sialome components, equivalent to 0.2 pairs of salivary glands, are analyzed on Coomassie Blue stained SDS-PAGE (lane 2), and by immunoblot analysis using rec-*Gmm* TSGF-2 antibodies (lane 3) and anti-*Gff* saliva antibodies (lane 4). Lane 1 shows molecular marker. * indicates TSGF-2 corresponding protein band.

We generated polyclonal rabbit antibodies against the recombinant (rec) *Gmm* Tsal1 (40 kD), TSGF-1 (55 kD) and TSGF-2 (60 kD) and used these antibodies for immunoblot analyses of the same amount of sialome extracts obtained from different tsetse species (Fig 4). Unlike anti-saliva antibodies generated through the natural fly bite, antibodies against *Gmm* recTsal1 and *Gmm* recTSGF-1 recognized the corresponding proteins from the sialomes of both *Morsitans* and *Palpalis* group flies (Fig 4A and 4B). In contrast, recTSGF-2 antibodies detected the corresponding 60KD protein from *Gmm* and *Gpg*, but not from *Gpd* (denoted by * in Fig 4C). Thus, although the *Gmm* TSGF-1 and TSGF-2 proteins are non-immunogenic when introduced to mice through the natural feeding route, the corresponding rec-proteins in association with adjuvant appear to be immunogenic in rabbits. The recTSGF-2 antibodies recognized the same protein in *Gpg* sialome (Fig 4D lane 3) that showed the strongest signal with anti-*Gff* saliva antibodies (Fig 4D lane 4) suggesting that the most immunogenic fraction in the *Palpalis* group sialome corresponds to TSGF-2.

### Anti-saliva antibodies in field cattle sera

**Fig 5:**
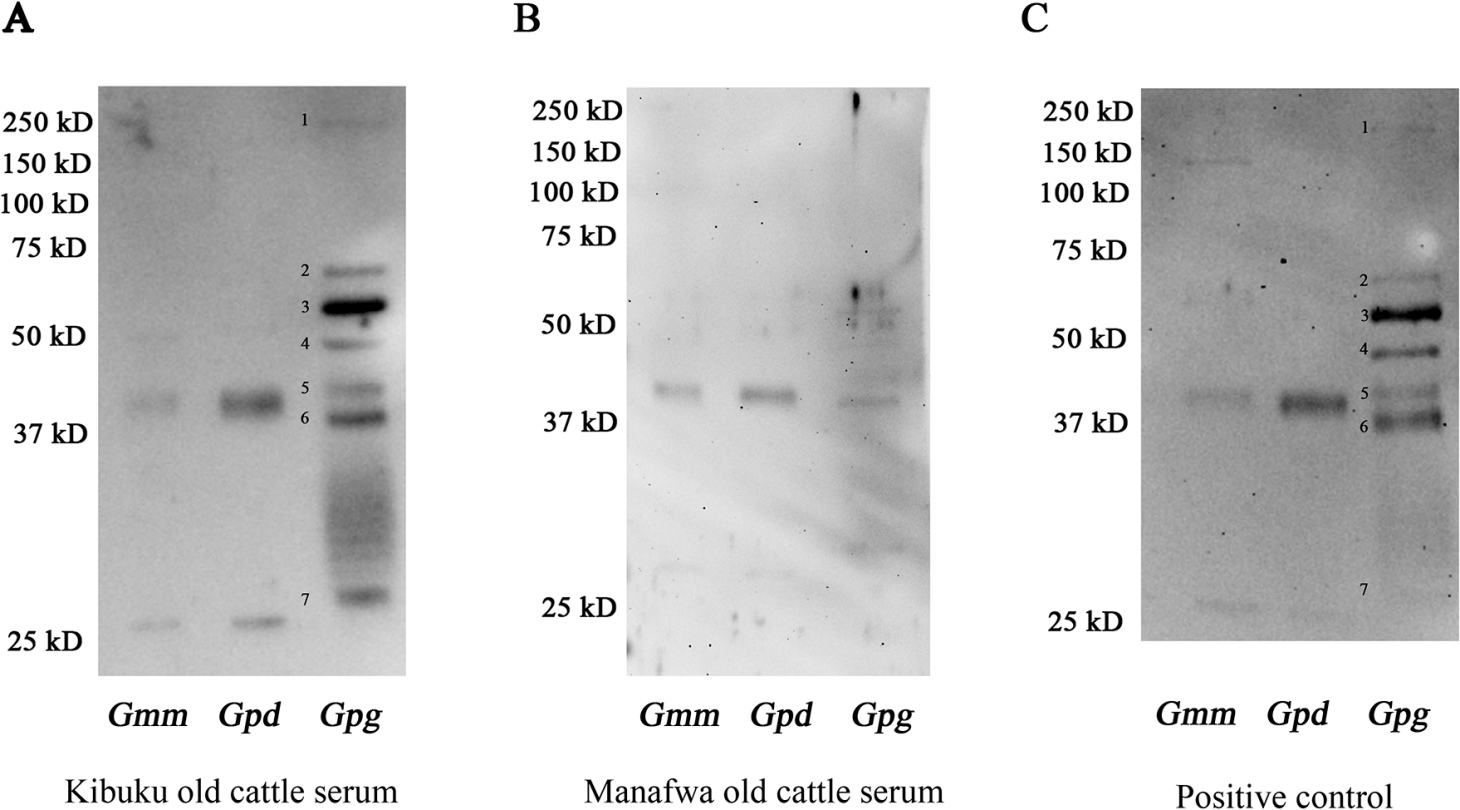
Immunoblot analysis of sialome proteins from *Gmm*, *Gpd* and *Gpg* using endemic cattle sera. Sialomes from *Gmm*, *Gpd*, and *Gpg* were analyzed with sera from (A) 8–12 year old cattle from Kibuku district, (B) 10–15 year old cattle from Manafwa district and (C) cattle used for *Gff* colony maintenance included as positive control.

To understand anti-saliva specific antibody responses in animals living under natural fly challenge in endemic areas, we obtained sera from young (less than 8 month old) and old cattle (aged 8–15 years) from the Kibuku and Manafwa areas of Uganda (S2 Table). In Kibuku, *Gff* is the predominant tsetse species, while in Manafwa *Gpd* is more abundant (Personal communication with Dr. Loyce Okedi, NaLIRRI). We also obtained positive control sera from cattle that were used to maintain the *Gff* colony in the insectary in Uganda, and commercial FBS was used as negative control. Immunoblotting showed that older cattle in both Kibuku and Manafwa regions contained antibodies that recognized only a few species-specific sialome proteins (Fig 5). The signal we detected from young cattle was weaker than that obtained from older cattle while no signal was detected with the negative control FBS (S4 Fig). Sera obtained from animals in either region identified two proteins of 25 KD and 40 KD in *Gmm* and *Gpd* sialomes, which based on size correspond to Antigen 5 and Tsal, respectively. The blotting signal of the 25 KD protein from *Gmm* or *Gpd* saliva was much weaker compared to the signal detected with the 40 KD band. Sera obtained from animals in Kibuku district reacted more strongly with the *Gpg* sialome, possibly reflecting the greater abundance of the *Palpalis* group flies in Kibuku. Sera obtained from cattle in Kibuku as well as *Gff* exposed cattle detected up to seven protein fractions in the *Gpg* sialome (Fig 5A and 5C, respectively), the strongest signal corresponding to TSGF-2 based on size (60 KD). In addition to the seven distinct bands, the blots showed a smear around 30KD (Fig 5A and S3A Fig), which was less pronounced with positive control sera (Fig 5C). Such a smear was also noted in a previous study where sera obtained from humans in *Gff* epidemic area were used to detect responses to *Gpg* and *Gff* sialomes [41]. The smear may result from degradation products or a saliva component unique to *Palpalis* group flies, such as the bacterial symbionts associated with SG tissue as also suggested by the previous study [41]. Thus, cattle living under constant tsetse challenge exhibited only a low response to a small fraction of the sialome components similar to the laboratory experimental system in mice. Furthermore, field studies further support that the anti-sialome responses in naturally exposed animals reflect a species complex-specific profile, and titers are related to the intensity and length of fly challenge as was also noted before [19].

### Analysis of *Gmm* ADGF gene family

**Fig 6:**
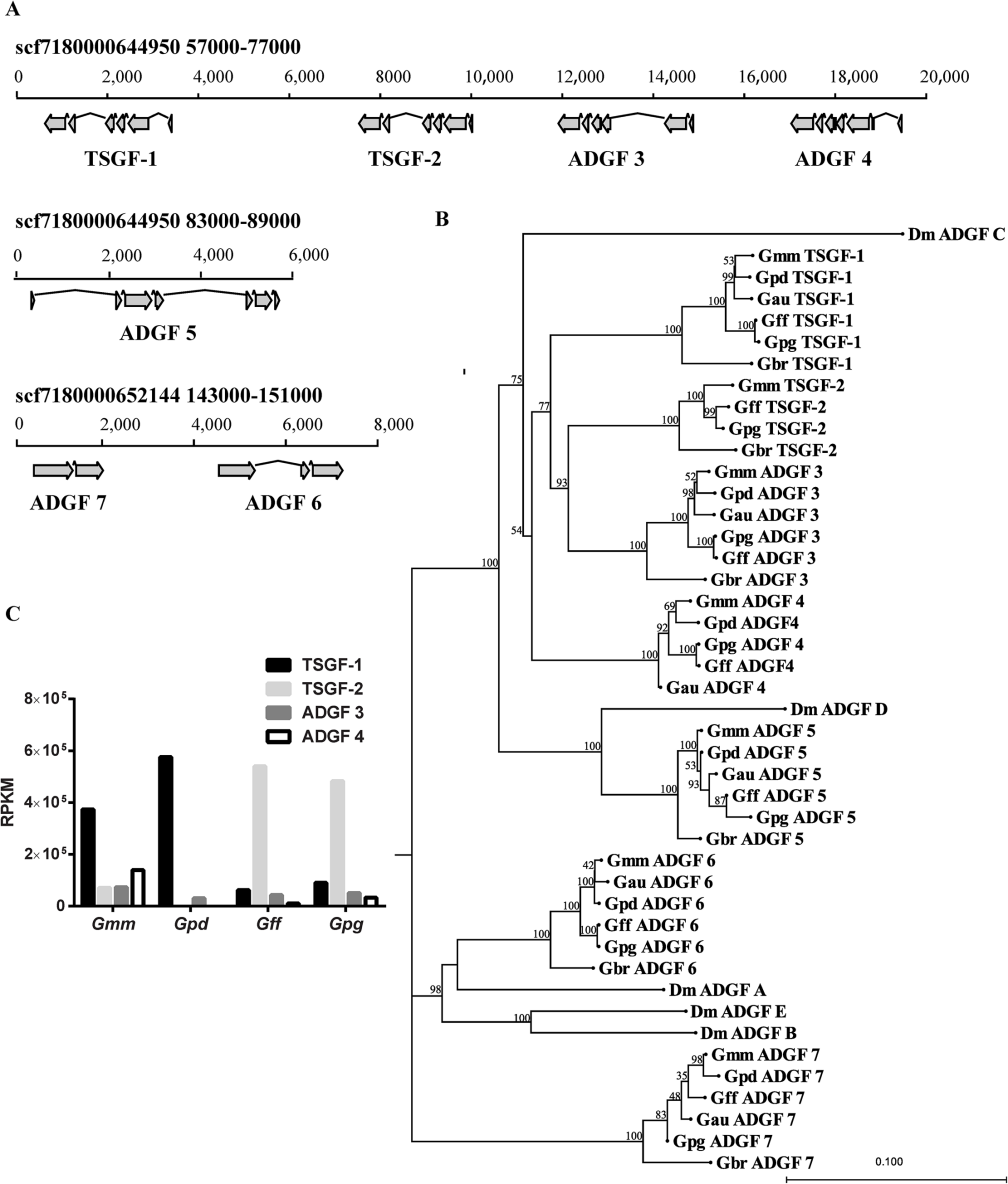
*Gmm* ADGF family gene organization and transcript abundance. (A): Chromosomal organization of *Gmm* ADGF family on genome scaffolds. (B) Phylogenetic tree showing the relatedness of the *Drosophila* spp. and *Glossina* spp. ADGF family members inferred from amino acid sequences. The scale bar represents 0.100 substitutions per site. The numbers on top of the branches indicate the bootstrap values. (C) The expression of the four members of the ADGF family of genes from *Gmm*, *Gpd*, *Gpg and Gff* salivary gland. The RNAseq based transcriptomes of salivary glands in different tsetse species were analyzed and the transcript abundance values are shown as RPKM.

We focused on the ADGF protein family because based on our immunoblotting data, this family of proteins do not appear to elicit a strong immune response despite being highly abundant in the *Gmm* sialome (Fig 3). Using *TSGF-1* and *TSGF-2* from *Gmm* and *ADGF A-E* from *Dm*, we searched the *Gmm* transcriptome and genome databases for related proteins, and identified 7 putative members for the ADGF family. We refer to the members of the ADGF family in tsetse as TSGF-1 and TSGF-2 and ADGF 3–7 [40, 42]. The genes encoding TSGF-1 and -2, and ADGF 3–5 are located on the same genomic contig spanning over a 32 kb region, while ADGF 6–7 are organized on the other scaffold (Fig 6A). With the exception of *ADGF-5*, which is abound 6000 bps, all members of the *ADGF* family are around 1500–3000 bps in size. Based on their genomic structure, *TSGF 1–2* and *ADGF 3–5* have 6 or 7 exons, while *ADGF-6* and -*7* have 3 and 2 exons, respectively (Fig 6A). The putative ADGF proteins are 485–544 aa in size, with the exception of ADGF-5 which encodes a shorter putative protein of 202 aa due to the presence of a premature stop codon. With the exception of ADGF-7, all putative ADGF proteins contain a secretory signal peptide (S3 Table). Nucleotide and deduced protein sequences were compared between the *Gmm* ADGF family members and *Drosophila* ADGFs (Table 1). Four members of the ADGF family, TSGF 1–2, and ADGF 3–4, showed the highest similarity, which ranged from 50–60% at the nucleotide level, and 40–50% at the protein level.

**Table 1:** Sequence alignment comparison of *Gmm* TSGF family with *Drosophila* ADGF family.

AA	*Gmm* TSGF 1	*Gmm* TSGF 2	*Gmm* ADGF 3	*Gmm* ADGF 4	*Gmm* ADGF 5	*Gmm* ADGF 6	*Gmm* ADGF 7*	*Dm* ADGF A	*Dm* ADGF C	*Dm* ADGF D
**NA**										
***Gmm* TSGF-1**		41.44	42.44	39.85	12.55	27.01	23.15	25.80	**40.47**	36.12
***Gmm* TSGF-2**	51.71		48.16	46.83	13.06	29.93	25.87	30.21	**46.23**	37.52
***Gmm* ADGF 3**	53.87	58.27		49.91	11.00	31.54	25.24	28.98	**43.27**	36.26
***Gmm* ADGF 4**	50.55	56.42	59.28		13.59	32.74	23.33	32.75	**50.37**	39.66
***Gmm* ADGF 5**	48.62	48.97	50.47	50.03		10.05	9.57	9.95	14.20	**26.28**
***Gmm* ADGF 6**	41.20	43.74	44.14	47.27	44.02		31.03	**57.85**	31.83	32.85
***Gmm* ADGF 7** *	38.33	40.12	39.46	39.52	39.74	42.20		**30.43**	25.39	27.61
***Dm* ADGF A**	39.21	41.90	42.60	42.75	40.96	**56.35**	**40.00**		31.45	33.98
***Dm* ADGF C**	**46.77**	**50.55**	**51.45**	**51.39**	45.68	42.61	38.42	43.23		42.00
***Dm* ADGF D**	45.55	46.28	46.93	46.00	**62.60**	42.95	37.41	42.59	51.39	

Note: Bold characters indicating the highest identity of TSGF with *Dm* ADGF sequences.

*: TSGF7 have a stop codon in 607–609 bp. Here the protein sequence included in the comparison is only 202 aa. But the nucleotide sequence is the whole transcripts corresponding to the ORF sequence of other TSGF and ADGFs in alignment.

### Analysis of ADGF family in *Glossina* species

We searched genome and transcriptome data available for multiple tsetse species in the *Morsitans* (*Gmm*, *Gpd* and *G*. *austeni* (*Gau*)), *Palpalis* (*Gpg and Gff*), and *Fusca* (*G*. *brevipalpis* (*Gbr*)) groups to compare the ADGF family in *Glossina* spp. (Listed in S4 Table). Phylogenic analysis of ADGF family proteins from different *Glossina* species together with members of the *Dm* ADGF family are shown in Fig 6B. Overall, the relationships among the ADGF family members from different tsetse species reflected the species phylogeny determined by ITS-2 analysis [43]. The *ADGF* genes from members of the same subgenus were more closely related with each other than those from another subgenus. The ADGFs from *Gbr* were consistently the most divergent among the five tsetse species analyzed, confirming the earlier separation of the *Fusca* subgenus in *Glossina* evolution. Phylogenetic analysis confirmed the close relatedness of tsetse TSGF-1, TSGF-2, ADGF-3 and ADGF-4 with *Dm* ADGF-C. The analysis also showed that tsetse ADGF-5 is related to *Dm* ADGF-D, tsetse ADGF-6 to *Dm* ADGF A, while tsetse ADGF-7 did not have a *Dm* homolog. Based on the sequence and phylogenetic relatedness along with co-localization on the genome, genes encoding TSGF 1–2 and ADGF 3–4 may represent a recent expansion in the genus *Glossina*. Interestingly, neither *Gpd* nor *Gau* genomes, encode a *TSGF-2* homolog based on genomic and transcriptomic analysis.

Analysis of the transcriptome data from *Gmm*, *Gpd*, *Gpg and Gff* indicated expression of only *TSGF 1*–2 and *ADGF 3–4* in the salivary gland tissue. Among these four *ADGF* subtypes, *TSGF-1* is the highest expressed gene in *Morsitans* group species (*Gmm* and *Gpd)*, corresponding to 57% of total *ADGF* expression in *Gmm*, and to 95% in *Gpd*, respectively. In *Gmm* salivary glands, *TSGF-2* and *ADGF 3–4* also have significant expression (7.0–13.9 RPKM), while in *Gpd*, which lacks *TSGF-2* genomic locus, there was little to no expression of *ADGF 3–4* (Fig 6C). In the *Palpalis* group (*Gpg* and *Gff*) salivary glands, the major expressed subtype was *TSGF-2*, corresponding to 83% in *Gff* and 74% in *Gpg* of total *ADGF* expression, respectively (Fig 6C). Despite sharing a common origin, these four genes display isotype specific expression profiles in the different tsetse species.

### TSGF-1 and TSGF-2 antibody effect on *Gmm* fitness

**Fig 7:**
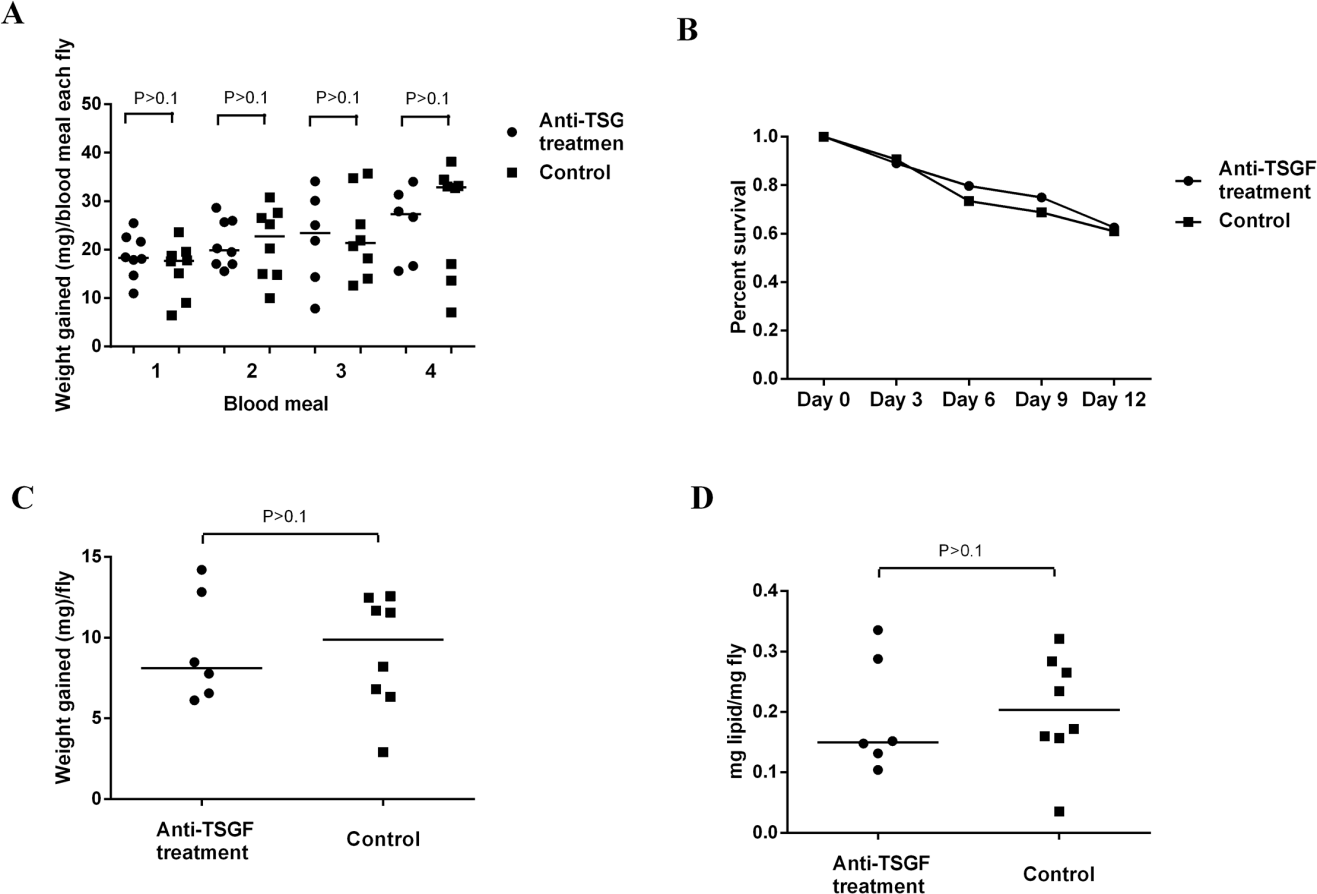
The feeding efficiency and survival of flies that had four bloodmeals on mice that received anti-recTSGF 1–2 antibodies. (A) Mean engorged blood meal weights of flies measured before and after each blood meal. Eight cages of flies were allowed to feed on mice at Day 2, Day 5, Day 8 and Day 11 after the mice received rabbit anti-recTSGF treatment. Cages were weighed before and after each blood meal. Each data point corresponds to one cage, which includes the average blood engorgement data for all alive flies in that cage (n = 8 flies per cage at the beginning). (B) Percent survival of flies maintained on rec-TSGF antibody treated and control mice, respectively. The first day they were exposed to mice is considered Day 0. Both groups had 64 flies at the beginning of the experiment. (C) Average weight change in each cage between Day 1 and Day 12. Each data point represents the average weight change in each cage. As one mouse in the TSGF antibody treated group died before the 3^rd^ blood meal, 6 cages of flies from the TSGF antibody treated group and 8 cages from the control group were measured here. (D): Lipid levels determined from flies 72 h after their 4th blood meal. Each data point represents the average of all alive flies in each cage (n = 6 cages in TSGF antibody treated group and n = 8 cages in control group). Statistical analyses were performed using the Mann Whitney test in the GraphPad Prism 6 software package. No significant difference were detected between the two groups (P>0.1).

Since our results indicated that *Gmm* TSGF proteins are largely invisible to the mice immune system when introduced upon tsetse bites, we reasoned that TSGF family may have some important function(s) during the blood feeding process. To test our hypothesis, we passively immunized mice with both the rec-TSGF-1 and rec-TSGF-2 antibodies, and included a control group that received pre-immune sera. We performed ELISA to ensure that antibody titers remained high in mice during the experimental period. We also confirmed that sera from mice that received the pre-immune sera did not cross react with rec-TSGF antigens (S5 Fig). We allowed teneral virgin male and female *Gmm* flies to receive four blood meals on the passively immunized mice and at the conclusion of the experiment evaluated flies for feeding efficiency and fitness effects by measuring engorgement, mortality and total lipid levels (Fig 7). *Gmm* fed on immunized mice showed slightly lower, but not statistically significant engorgement, when measured after the second and fourth blood meals in comparison to the control groups, 12.6% versus 17.0%, respectively (Fig 7A). The mortality rates between the experimental and control groups were similar during the experimental duration, 62.5% in flies that received the anti-TSGF antibodies versus 60.9% in the control group, respectively (Fig 7B). Over the experimental period, flies maintained on control mice had slightly higher total weight change (9.9 mg) than those maintained on passively immunized mice (8.1 mg), although this difference was not statistically significant (P>0.1) (Fig 7C). The total lipid levels in the control group (0.20 mg) also showed a slightly higher but not significant (P>0.1) change from experimental flies (0.15 mg) (Fig 7D). Thus, exposure of *Gmm* for two weeks to mice that had anti-TSGF-1 and -2 antibodies resulted in a slight detriment to their blood feeding ability as reflected by compromised digestion, lower weight gain and less nutritional resource availability although these results were not statistically significant.

## Discussion

Blood feeding insects transmit disease agents to humans and animals worldwide. For successful blood feeding, insect saliva contains a variety of molecules (termed sialome) with functions important for evading the hematological and immune system of the vertebrate host at the bite site [29]. For long-term success, it is important for the sialome components not to trigger strong host immune responses, which can otherwise interfere with the insects’ feeding ability. Our results from four different tsetse species that belong to two different species complexes of *Glossina* confirm that the major abundant sialome proteins do not induce high immunogenic responses in both laboratory mice and in cattle living under tsetse challenge in endemic areas.

Our immunoblotting results show that the few sialome proteins with immunogenic potential when introduced through the natural feeding route show limited cross-reactivity between different fly species, particularly among those that belong to different tsetse subgenera. We evaluated the potential function(s) of one of the most abundant sialome proteins, TSGF-1 and TSGF-2, which belong to ADGF family of proteins with adenosine deaminase activity. Our data suggest that limited exposure of flies to mice that have passively received anti-recTSGF 1–2 antibodies results in a negative trend on tsetse fitness parameters. Both the abundance and the specific members of the ADGF family of proteins expressed in the sialome of different tsetse species may influence tsetse’s long-term fitness, host preference and disease transmission characteristics.

Studies on the proteins present in the sialomes of different blood feeding insects provide fundamental information for saliva research with practical applications for disease control [44, 45]. Our analysis of the major sialome proteins from four tsetse species indicates that these proteins are fast evolving and that flies belonging to different species complexes display similar protein profiles and species-specific immunogenicity in experimental mice. In *Gmm*, Sgp3, Tsal and Antigen 5 have higher immunogenicity than the other abundant proteins, such as 5’ nucleotidase and TSGF family (Figs 2 and 3). However in *Gff*, proteins that belong to the ADGF family display the highest immunogenicity potential.

Immunogenicity of sialome proteins, and cross reactivity of saliva mediated immunological responses among different vector sub-species have been analyzed in several other blood-feeding insects [29, 46–48]. In the mosquitoes *Aedes communis*, *Aedes aegypti* and *Anopheles stephensi*, most of the saliva antigens appear to be species-specific and only weak cross-reactivity is observed with heterologous immune sera [47]. The *Leishmania* spp. sand fly host species *Phlebotomus papatasi*, *Phlebotomus sergenti*, and *Lutzomyia longipalpis* also have unique saliva protein profiles, and sera from mice exposed to these three species specifically do not exhibit extensive cross reactivity [48]. Studies in different tsetse species similarly reported varying serological responses to salivary proteins [19, 21, 22, 49]. Caljon et al. evaluated host antibody responses to the highly immunogenic family of the endonuclease-like Tsal proteins using mice previously exposed to multiple tsetse species and rec Tsal1 proteins. Based on the cross reactivity they observed, they suggest that detection of anti-rTsal1 IgGs could be a promising serological indicator of tsetse fly presence [49]. The anti-saliva antibodies we generated in this study represent a low fly challenge, and our immunoblotting analysis did not detect obvious cross reactivity for Tsal1 proteins between the species we analyzed in the different tsetse subgenera. This difference may reflect the varying sensitivity associated with the different methods we used for detecting the immunological responses, but also may be related to the varying intensity of fly challenge the experimental mice were subjected to. In the study by Somda et al., the anti-saliva responses in experimental cattle were shown to depend on the number and frequency of fly challenge. They reported that *G*. *m*. *submorsitans (Gms)* saliva showed a broad cross reactivity with sera of cattle exposed to different tsetse fly species. But sera from cattle exposed to *G*. *m*. *submorsitans (Gms)* exhibited weaker cross reactivity to *Gpg* saliva than sera from animals exposed to *Gpg* bites [19].

The antisera we developed in the laboratory could differ from the sera generated in animals in Africa that are naturally bitten by tsetse flies. The composition of the saliva we used from the cultured tsetse fly lines might vary especially since the colony flies we tested are maintained on artificial feeding systems that do not rely on the anticoagulation functions of saliva. In fact, previous research on sand flies have found that laboratory fly saliva can induce better protection against *Leishmania* infection than saliva from wild-caught or recently colonized sand flies [29, 50]. Moreover, the duration and frequency of the fly bites the animals in Africa are naturally exposed to also differ from the laboratory experimental conditions. Furthermore, the sera obtained from native animals would contain responses to other blood sucking insect bites the animals are exposed to. To investigate the immunological responses of animals living under natural tsetse challenge, we compared the cross-reactivity of sera collected from cattle in two districts of Uganda where tsetse species distribution varies. We used the closely related *Gpg* for our analysis with endemic cattle sera as we did not have access to *Gff* fly saliva during the course of our later studies due to colony collapse. We also used *Gmm* and *Gpd* for our analysis with natural sera. Despite these potential variations, generally, our analysis with endemic cattle sera confirmed our findings with experimental mice in that sialomes from different fly species generate varying immune signatures. The cattle sera from Kibuku district, where the *Palpalis* group species *Gff* is the dominant tsetse species, had stronger interaction with the closely related *Gpg* sialome than with either *Gpd* or *Gmm*. In contrast, cattle sera from Manafwa area, where *Gpd* is present, showed stronger interaction with *Gmm* and *Gpd* sialomes. This would be expected as *Morsitans* group flies have strong preference for cattle, thus it is likely that immune responses of the cattle would reflect this bias. Among the sialome proteins, *Gpg* TSGF-2 showed the strongest signal with sera from Kibuku area, while the same sera could not detect TSGF-2 from *Gmm* and *Gpd* by immunoblot analysis. Beyond the presence of the *Palpalis* group flies in Kibuku area, the strong response we detected with TSGF-2 may also reflect the varying abundance of this protein in different species sialomes. In concordance, the transcriptomics analysis indicates that *TSGF-2* is expressed at low levels in *Gmm*, and is missing from *Gpd* genome while *TSGF-2* is expressed at high levels in *Gpg* (Fig 6C). Analysis of sera from cattle aged 8–15 years showed a stronger immunological response to specific sialome antigens than cattle less than 8 months old, confirming that anti-saliva antibody titers increase over time related to frequency of fly challenge. Nevertheless, the magnitude of these responses were quite restricted even in older cattle that likely received many tsetse bites, suggesting that cattle repeatedly exposed to bites may eventually gain tolerance to the bites of those species. In previous works, human and cattle in tsetse epidemic and free areas were also compared to illustrate that anti-saliva antibody titers vary by fly bites in rainy and dry seasons where tsetse densities fluctuate [19, 51].

Immunogenic components of saliva have been exploited as biomarkers for exposure to different arthropod bites, including ticks, sandflies and mosquitoes [45, 46, 52, 53]. The potential use of tsetse saliva as a biomarker of exposure has also been investigated using human and cattle sera [19–21]. The saliva antigens in *Gpg* were analyzed to detect human exposure to tsetse flies in West Africa [21]. Sera from humans in Uganda scored positive for saliva-specific IgGs by ELISA detection, and against recombinant *Gmm* Tsal proteins by immunoblotting [51]. Tsal protein has been reported in previous works to be a good antigen to detect human and animal exposure to tsetse bites [49, 51]. In our study, analysis of the cattle sera from both tsetse epidemic areas commonly recognized Tsal proteins in *Gmm*, *Gpd* and *Gpg*, which also confirms previous findings. While Tsal protein responses might be good for evaluating the risk for general tsetse exposure, TSGF-2 would be particularly useful for detecting exposure to *Palpalis* group fly challenge based on our immunoblot analysis. In addition a TSGF-1 specific peptide corresponding to aa 18–43 has been proposed as a good biomarker of tsetse exposure in *Gpg* epidemic area [22].

The ADGF family in tsetse has seven members, but only TSGF 1–2 and ADGF 3–4 are preferentially expressed in salivary gland. These four related genes are co-localized in the *Gmm* genome, indicating a recent gene duplication event. All four genes are related to *Dm* ADGF-C, which is actually not a highly expressed subtype in *Drosophila* spp. [54]. In *Drosophila* spp. brain and salivary glands, the isotype ADGF-D is expressed, which is more closely related to tsetse ADGF-5 [54]. It is possible that the expansion of TSGF 1–2 and ADGF 3–4 may have evolved with the blood feeding diet of tsetse, suggesting that ADGFs may play important function(s) for tsetse’s blood feeding process. The transcriptome data indicated that the four members of the ADGF family genes are expressed at varying levels in the salivary glands of different tsetse species. In the *Morsitans* group, *Gmm* and *Gpd*, *TSGF-1* is the highest expressed member of the family, while in the *Palpalis* group, *Gff* and *Gpg*, *TSGF-2*, is the highest expressed member. The differences in the abundance of varying ADGF isotypes in the sialomes of different species may contribute to the varying saliva immunogenicity we detected but also may influence their ability to feed on specific hosts or reflect adaptation to their preferential hosts. While the *Palpalis* group flies feed preferentially on humans and are efficient vectors of human-infective trypanosomes, the *Morsitans* group tsetse prefer feeding on ungulates and are more efficient vectors of the animal disease causing trypanosomes [13, 55]. Analysis of transcriptomes from normal and parasitized salivary glands of *Gmm* indicate that the expression of all four *ADGF* genes are significantly down regulated in infected flies [37]. It remains to be seen whether the variation in the abundance of the different ADGF isotypes we noted in the different species complexes may influence the varying vector competence noted in the different tsetse host species [56].

Adenosine deaminase (ADA) deficiency is lethal in *Drosophila* [42]. Studies have shown that in *Drosophila* both ADGF-C (the most closely related protein family member in *Dm* to the four tsetse ADGF family proteins) and ADGF-D are mitogenic *in vitro*, stimulating cell proliferation by depleting extracellular adenosine [54, 57]. Our previous analysis had shown that *Gmm* saliva has highest ADA activity, with *G*. *p*. *palpalis* (*Gpp*) having much less, and *Gau* no ADA activity [13]. *Gpp* is closely related to *Gpg* and *Gff* in the *Palpalis* subgenus, while *Gau* is closely related to *Gpd*, and both *Gau* and *Gpd* have lost the *TSGF-2* locus. It is possible that differences in ADA activity levels in the different tsetse species saliva may influence blood feeding processes and vector competence traits. Adenosine deaminase was found in several other blood feeding insects including sand fly and mosquitoes [13, 58–61]. In mosquitoes, ADA activities were detected in the saliva of *Culex pipiens quinquefasciatus* (vector of avian malaria and West Nile virus) and *A*. *aegypti* (vector of Dengue and Yellow Fever viruses), but not in the Anopheline mosquito *Anopheles gambiae* (vector of human malaria) [58]. In the sand fly, ADA activity has been detected in *L*. *longipalpis* and *Phlebotomus duboscqi* saliva, but not in *P*. *papatasi*, *Phlebotomus argentipes*, *Phlebotomus perniciosus* or *Phlebotomus ariasi* [61].

To understand the functional role of ADGF protein family in tsetse, we maintained flies on mice that were passively transferred anti-recTSGF-1 and recTSGF-2 IgGs. Although overall the blood intake, weight and total lipid levels in the anti-TSGF blood meal receiving group were lower than the control after four blood meals, these differences were not statistically significant between the two groups. In Caljon et al’s study, anti-saliva immunized mice also showed no negative effect on tsetse fly blood feeding efficiency and survival [51]. It is possible that the four blood meals the flies received during our study were not sufficient to cause a detrimental effect on major host fitness parameters. Furthermore the TSGF-1 and TSGF-2 polyclonal sera we used may not block ADGF 3 and -4 specified activities, which may compensate due to potential functional redundancies. Finally, it is possible that passive transfer of rabbit IgGs to mice may mount an anti-rabbit immunoglobulin response, resulting in a serum sickness reaction later in the process. Thus, formation of mouse anti-rabbit immunoglobulin immune complexes late in the experiment may have reduced the available amount of anti-tsetse protein antibodies. Thus, future studies would focus on experiments where anti-recTSGF mice IgGs may be tested using the same bioassay. Previous studies have reported that adenosine in saliva can help initiate perception of pain in vertebrates, and also have vasodilatory, anti-platelet aggregation and lymphocyte-immunosuppressive activities [62] while inosine can potently inhibit production of inflammatory cytokines [60]. Adenosine deaminase activity in insect saliva may play a role in regulating the concentration of saliva adenosine and inosine levels. It is possible that because the mice used in our study were anaesthetized while exposed to fly bites, the mice may not feel the itch or pain caused by saliva adenosine. So the effect of blocking ADA activity by antibodies may not cause host behaviors that might influence the amount of blood the flies are able to take up at each feeding. The expression levels of the different TSGF proteins in saliva of different tsetse species vary. Furthermore, TSGF proteins expressed in the different tsetse species vary in their immunogenicity as also indicated by studies reported by Dama E et al., [21]. It remains to be seen whether these differences influence host feeding preferences of the different tsetse species.

In summary, both the composition and the immunogenicity potential of the sialome proteins vary in the different tsetse species groups. In *Gmm*, the major immunogenic proteins are TAg5, Tsal1/Tsal2, and Sgp3, while in *Gpg* the immunogenic proteins include the Adenosine Deaminase Growth Factor (ADGF) family. We show that of the seven members of this family, only 4 are expressed at varying levels in the salivary gland of different tsetse species. The different ADGF subtypes expressed in the different tsetse species may contribute to the varying levels of immunogenicity this family displays. The relationship of ADGF family proteins with host vector competence traits, as well as their potential role as bio-marker of exposure to the different tsetse species complexes merits further investigations.

## Supporting Information

S1 Table Age and geographic information of endemic cattle analyzed in the study. (DOCX)

S2 Table LC-MS/MS Protein Identification. (DOCX)

S3 Table *Gmm* ADGF protein family. (DOCX)

S4 Table ADGFs among the different tsetse species. (XLSX)

S1 Fig SDS PAGE analysis of saliva and salivary gland samples. (A) Secreted (Lanes 1–4) and non-secreted (lane 5–8) salivary gland proteins from *Gmm*, *Gpd*, *Gff* and *Gpg*. (B) SDS PAGE analysis of saliva from *Gmm*, *Gpd*, *Gff* and *Gpg*. Showing replicate experiments.
(TIF)

S2 Fig Replicating experiments of the protein immunogenicity detection of the saliva from *Gmm*, *Gpd*, *Gff* and *Gpg*. The immunoblots were probed with anti-*Gmm* saliva antisera from different mice.
(TIF)

S3 Fig Native PAGE and immunoblot analysis of saliva proteins from *Gmm*, *Gpd* and *Gpg*. (A) Native PAGE of saliva proteins from *Gmm*, *Gpd* and *Gpg*. (B) Immunoblot probed by *Gmm* saliva antibodies. (C) Immunoblot probed by *Gff* saliva antibodies.
(TIF)

S4 Fig Immunoblots detecting interactions between cattle serum collected in Eastern Uganda with saliva from *Gmm*, *Gpd*, and *Gpg*. The blots were probed with sera from (A) calves aged 2–4 months from Kibuku (B) calves aged 3–8 months from Manafwa and (C) commercially available FBS.
(TIF)

S5 Fig Course of the anti-TSGF antibody titer in passive-immunized mice. (A): TSGF-1 antibody titer and (B): TSGF-2 antibody titer detected by ELISA. Mouse serums were collected on Day 1 and Day 12 post antibody injection. Data were represented as ΔOD 450.
(TIF)
