# Supplementary material for: Immunogenicity and Serological Cross-Reactivity of Saliva Proteins among Different Tsetse Species
Source: PLoS Negl Trop Dis. 2015 Aug 27;9(8):e0004038. doi: 10.1371/journal.pntd.0004038 (PMC4551805; doi:10.1371/journal.pntd.0004038)
Supplement: S1 Table — (DOCX) [file pntd.0004038.s001.docx]

**S1 Table. The age and geographic information of cattle.**

|  | **District** | **Country** | **Sample No.** | **Age** |
| --- | --- | --- | --- | --- |
| Group 1 | Kibuku | Kibuku | 60 | 10 years |
|  |  |  | 66 | 8 years |
|  |  |  | 77 | 8 years |
|  |  |  | 88 | 10 years |
|  |  |  | 90 | 10 years |
|  |  |  | 96 | 12 years |
| Group 2 | Kibuku | Kibuku | 23 | 4 months |
|  |  |  | 78 | 2 months |
|  |  |  | 80 | 3 months |
|  |  |  | 81 | 2 months |
|  |  |  | 89 | 3 months |
| Group 3 | Manafwa | Bubolu | 10 | 13 years |
|  |  |  | 29 | 14 years |
|  |  |  | 35 | 15 years |
|  |  |  | 42 | 12 years |
|  |  |  | 72 | 11 years |
| Group 4 | Manafwa | Bubolu | 5 | 5 months |
|  |  |  | 16 | 8 months |
|  |  |  | 33 | 3 months |
|  |  |  | 46 | 6 months |
|  |  |  | 54 | 8 months |
