# Supplementary material for: Immunogenicity and Serological Cross-Reactivity of Saliva Proteins among Different Tsetse Species
Source: PLoS Negl Trop Dis. 2015 Aug 27;9(8):e0004038. doi: 10.1371/journal.pntd.0004038 (PMC4551805; doi:10.1371/journal.pntd.0004038)
Supplement: S2 Table — (DOCX) [file pntd.0004038.s002.docx]

**S2 Tabel. LC-MS/MS Protein Identification**

**A: Identification of major immunogenic candidate proteins**

| **Score** | **Expectation** | **Protein ID** | **Protein Name** | **MW** | **% Coverage** | **empai** |
| --- | --- | --- | --- | --- | --- | --- |
| 2290 | 0 | gi\|8927464 | Tsal1 protein precursor [Glossina morsitans morsitans] | 45585 | 77.4 | 74.62 |
| 1819 | 0 | gi\|289740591 | Tsal2 protein precursor [Glossina morsitans morsitans] | 43928 | 69.8 | 24.41 |
| 1739 | 0 | gi\|125901748 | Tsal2 form A [Glossina morsitans morsitans] | 43974 | 69.1 | 27.2 |
| 1469 | 0 | gi\|126143295 | 5' nucleotidase [Glossina morsitans morsitans] | 100140 | 26.2 | 3.19 |
| 1428 | 0 | gi\|8927462 | antigen 5 precursor [Glossina morsitans morsitans] | 28906 | 72.2 | 132.08 |
| 832 | 9.20E-77 | gi\|14488055 | 5'-nucleotidase-related protein [Glossina morsitans morsitans] | 62025 | 33.3 | 2.04 |

**B: Identification of major non-immunogenic candidate proteins**

| **Score** | **Expectation** | **Protein ID** | **Protein Name** | **MW** | **% Coverage** | **empai** |
| --- | --- | --- | --- | --- | --- | --- |
| 2496 | 0 | gi\|289739673 | salivary gland growth factor-1 precursor [Glossina morsitans morsitans] | 56555 | 63.6 | 20.95 |
| 1175 | 4.80E-111 | gi\|289743375 | 5'-nucleotidase family salivary protein [Glossina morsitans morsitans] | 62023 | 37.5 | 3.1 |
| 1108 | 2.40E-104 | gi\|289742689 | adenosine deaminase-related growth factor C [Glossina morsitans morsitans] | 62162 | 44.3 | 1.43 |
| 831 | 1.30E-76 | gi\|5817646 | salivary gland growth factor-2 [Glossina morsitans morsitans] | 58186 | 32 | 1.58 |
| 611 | 1.40E-54 | gi\|289742693 | salivary secreted adenosine [Glossina morsitans morsitans] | 41195 | 22.1 | 2.4 |
