# Supplementary material for: Immunogenicity and Serological Cross-Reactivity of Saliva Proteins among Different Tsetse Species
Source: PLoS Negl Trop Dis. 2015 Aug 27;9(8):e0004038. doi: 10.1371/journal.pntd.0004038 (PMC4551805; doi:10.1371/journal.pntd.0004038)
Supplement: S3 Table — (DOCX) [file pntd.0004038.s003.docx]

| **Protein name** | **Protein sequence size (aa)** | **Signal peptide (aa)** | **Exon number** |
| --- | --- | --- | --- |
| TSGF 1 | 494 | 21 | 6 |
| TSGF 2 | 506 | 19 | 6 |
| ADGF 3 | 506 | 22 | 6 |
| ADGF 4 | 535 | 22 | 7 |
| ADGF 5 | 202 | 23 | 7 |
| ADGF 6 | 544 | 20 | 3 |
| ADGF 7 | 485 | No | 2 |

**S3 Table. *Gmm* ADGF protein family.**
