# Supplementary figures and images for: Immunogenicity and Serological Cross-Reactivity of Saliva Proteins among Different Tsetse Species
Source: PLoS Negl Trop Dis. 2015 Aug 27;9(8):e0004038. doi: 10.1371/journal.pntd.0004038 (PMC4551805; doi:10.1371/journal.pntd.0004038)

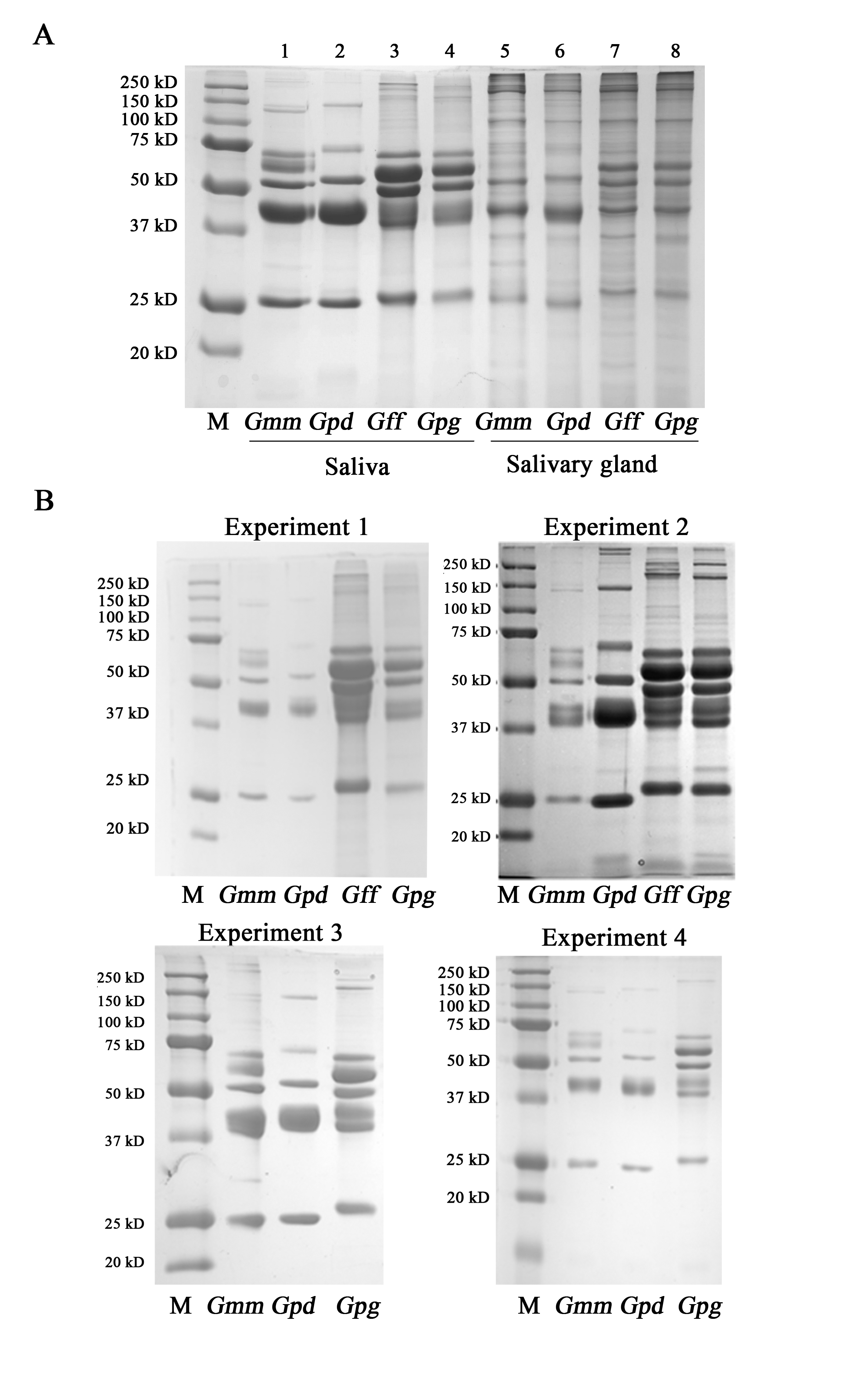

Supplement: S1 Fig — (A) Secreted (Lanes 1–4) and non-secreted (lane 5–8) salivary gland proteins from Gmm, Gpd, Gff and Gpg. (B) SDS PAGE analysis of saliva from Gmm, Gpd, Gff and Gpg. Showing replicate experiments. (TIF) [file pntd.0004038.s005.tif]

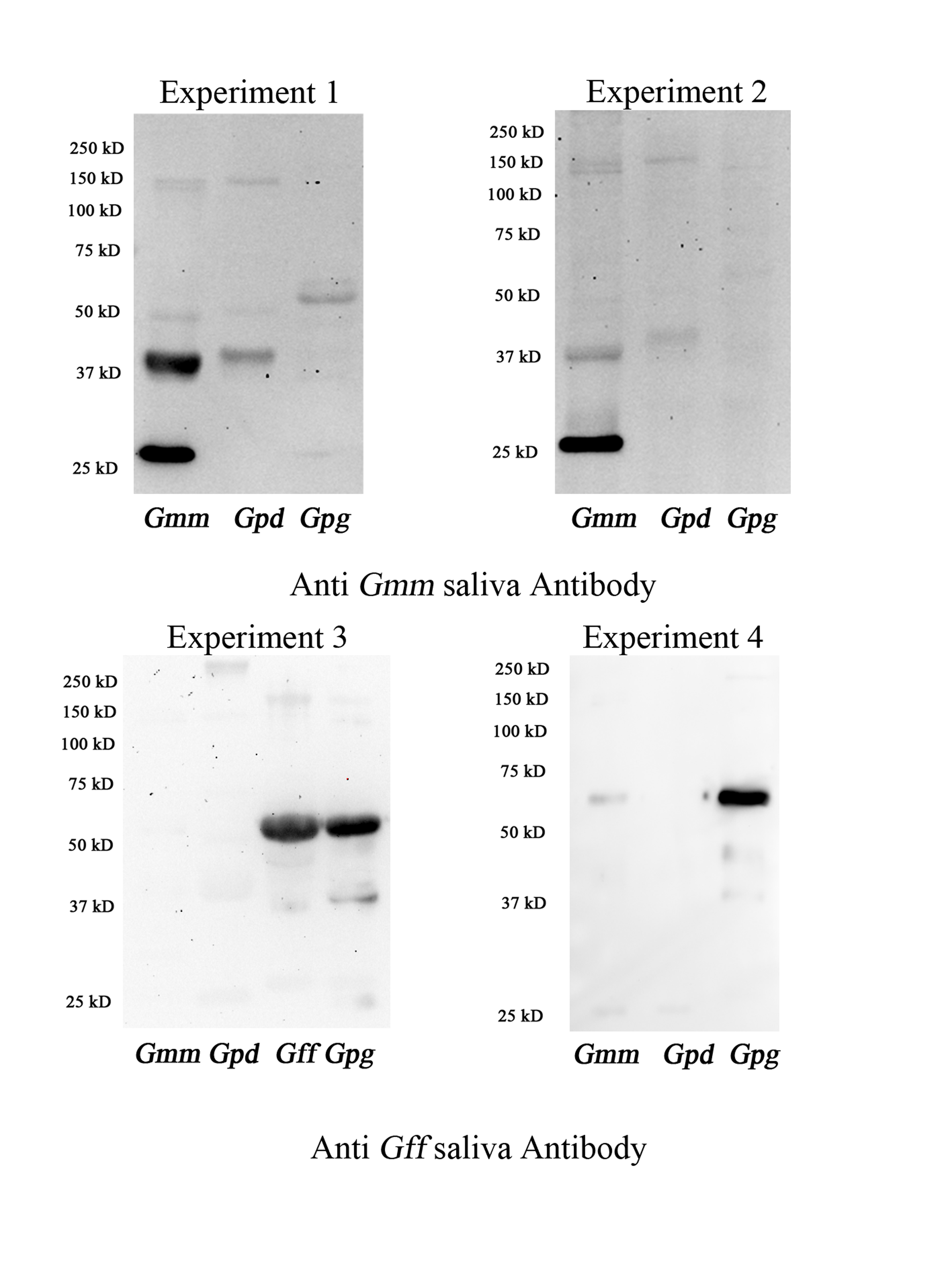

Supplement: S2 Fig — The immunoblots were probed with anti-Gmm saliva antisera from different mice. (TIF) [file pntd.0004038.s006.tif]

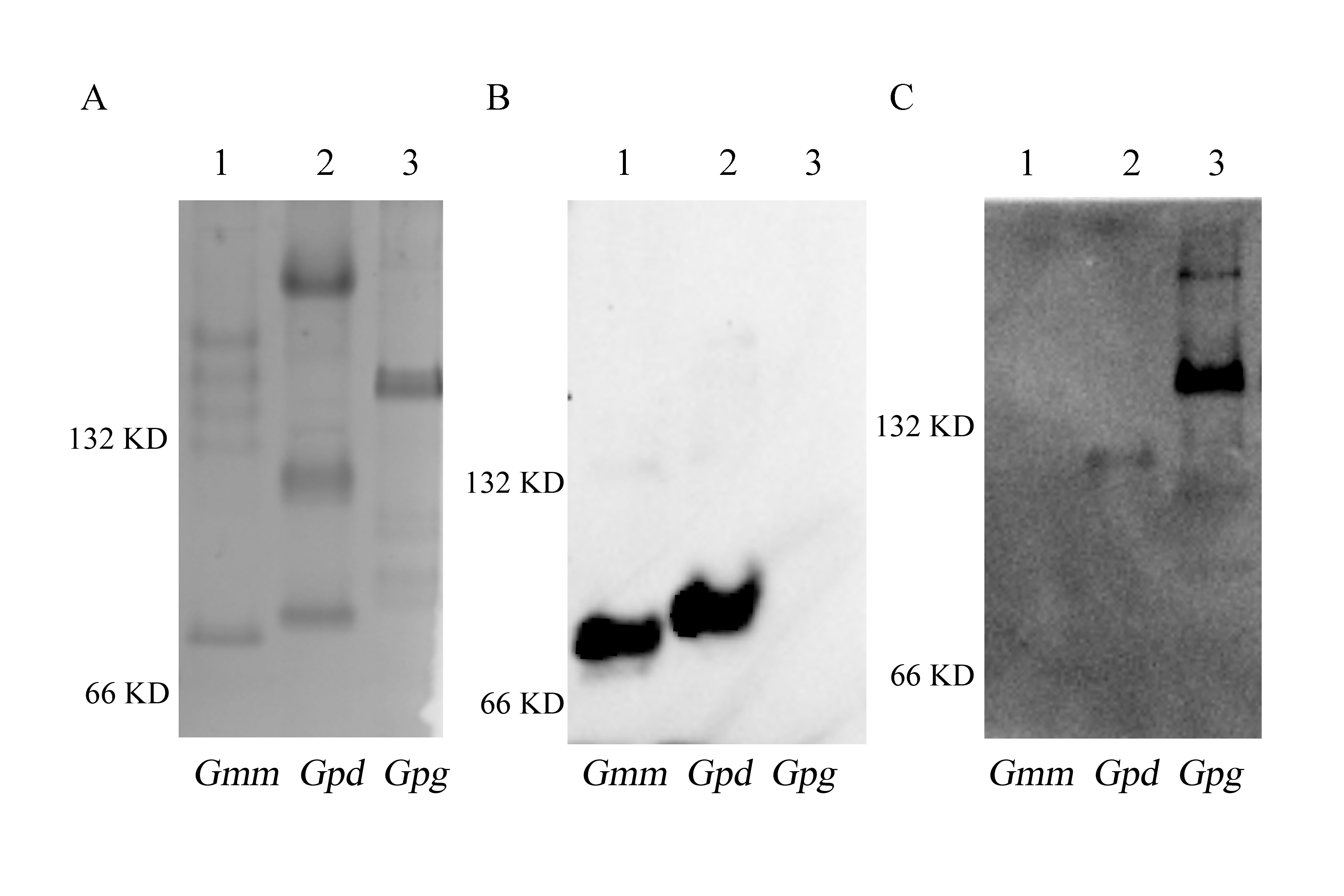

Supplement: S3 Fig — (A) Native PAGE of saliva proteins from Gmm, Gpd and Gpg. (B) Immunoblot probed by Gmm saliva antibodies. (C) Immunoblot probed by Gff saliva antibodies. (TIF) [file pntd.0004038.s007.tif]

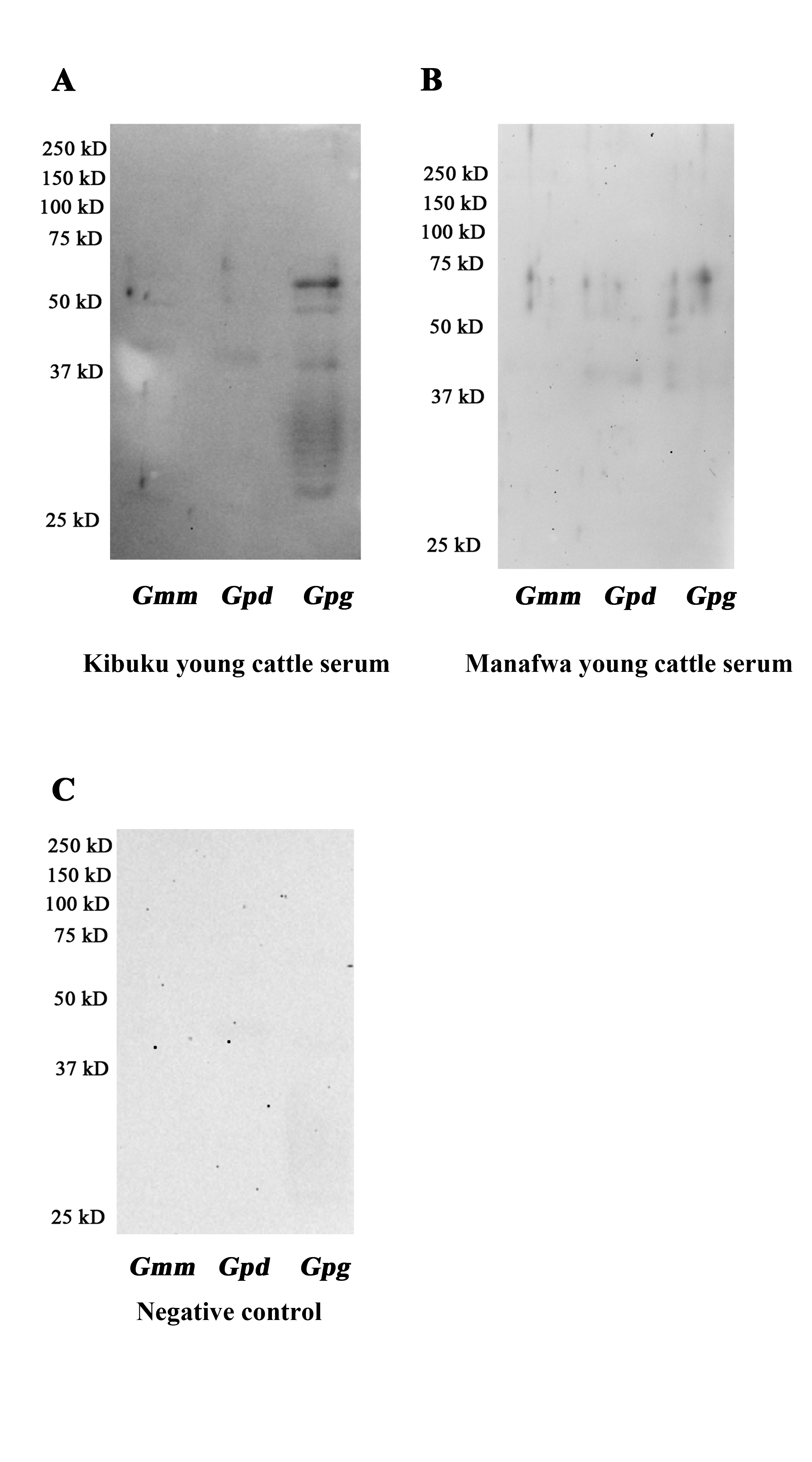

Supplement: S4 Fig — The blots were probed with sera from (A) calves aged 2–4 months from Kibuku (B) calves aged 3–8 months from Manafwa and (C) commercially available FBS. (TIF) [file pntd.0004038.s008.tif]
